# Mechanical Properties of the Extracellular Environment of Human Brain Cells Drive the Effectiveness of Drugs in Fighting Central Nervous System Cancers

**DOI:** 10.3390/brainsci12070927

**Published:** 2022-07-15

**Authors:** Mateusz Cieśluk, Katarzyna Pogoda, Ewelina Piktel, Urszula Wnorowska, Piotr Deptuła, Robert Bucki

**Affiliations:** 1 Department of Medical Microbiology and Nanobiomedical Engineering, Medical University of Białystok, PL-15222 Białystok, Poland; mticv1@gmail.com (M.C.); u.wnorowska@gmail.com (U.W.); piotr.deptula@umb.edu.pl (P.D.); 2 Institute of Nuclear Physics Polish Academy of Sciences, PL-31342 Kraków, Poland; katarzyna.pogoda@ifj.edu.pl; 3 Independent Laboratory of Nanomedicine, Medical University of Białystok, PL-15222 Białystok, Poland; ewelina.piktel@wp.pl

**Keywords:** rheology, brain, extra cellular matrix, mechanical properties, glioblastoma

## Abstract

The evaluation of nanomechanical properties of tissues in health and disease is of increasing interest to scientists. It has been confirmed that these properties, determined in part by the composition of the extracellular matrix, significantly affect tissue physiology and the biological behavior of cells, mainly in terms of their adhesion, mobility, or ability to mutate. Importantly, pathophysiological changes that determine disease development within the tissue usually result in significant changes in tissue mechanics that might potentially affect the drug efficacy, which is important from the perspective of development of new therapeutics, since most of the currently used in vitro experimental models for drug testing do not account for these properties. Here, we provide a summary of the current understanding of how the mechanical properties of brain tissue change in pathological conditions, and how the activity of the therapeutic agents is linked to this mechanical state.

## 1. Introduction

The extracellular matrix (ECM) is a three-dimensional network of macromolecules that acts as a substrate and physicochemical environment, in which cells grow and proliferate [1,2,3,4]. Notably, ECM is characterized by a highly dynamic structure that is continuously reconstructed, either non-enzymatically or enzymatically, and its molecular components undertake a variety of post-translational changes. Although the main biomacromolecules of ECM are collagens, fibrins, laminins, proteoglycans, glycosaminoglycans, and hyaluronic acid, each tissue and organ has its own ECM, which differentiates in composition and structure from the ECMs of other tissues and is constantly changing and remodeling in response to different stimuli [3,5,6]. Importantly, the ECM’s topological, physical, and biochemical makeup is not just tissue specific, but also shows high heterogeneity. For instance, ECM receptors such as discoidin domain receptors, integrins, and syndecans, which are present in variable levels across the tissue ECMs, are involved in cell attachment to the ECM [7,8,9], which further determines not only cell migration across this matrix [9], but also network density and its crossing. Additionally, by binding growth factors (GFs) and engaging with cell-surface receptors to activate signal transduction and govern gene transcription, the ECM guides the vital morphological structure and physiological function. Most crucially, ECM aids with protection through a buffering action that sustains extracellular homeostasis and retention of water through these physical and biochemical characteristics and creates each organ’s mechanical and biochemical parameters, such as elasticity and compressive and tensile strength [3,10,11]. Notably, the biomechanical and biochemical, organizational, and protective features of the ECM in a particular tissue can vary considerably, not only from one tissue to the next, but even within a single tissue, as well as from one physiological state to the next (for instance, normal versus cancerous tissue) [3,11]. In particular, the latter observation has a considerable clinical value, since the content and structure of ECM-like surfaces have a considerable impact on cell growth, differentiation, and cellular characteristics, which might promote oncogenesis, as was partially confirmed by some previous studies [12,13]. For this reason, researchers have been progressively recognizing and characterizing the ECM’s crucial role and its impact on nanomechanical properties of tissues in homeostasis and illness [14]. Currently, the most common methods to culture brain cells in vitro are to grow them on poly-lysine (-D or -L), collagen or hyaluronic acid-coated cell culture plates, but this approach completely ignores and experimentally eliminates the effect of the mechanical properties of the natural environment of brain on the cell’s biological response [3,15,16,17,18,19]. Numerous bio-inspired models for cell culture have been established during the last two decades, based mainly on functionalized, elastic polyacrylamide hydrogels, to reproduce the mechanical environment of the tissue and to determine how mechanical stimuli can influence cell activity. Nevertheless, an ever-growing amount of evidence demonstrates that viscous tissue properties (e.g., viscous dissipation) may potentially affect cellular response to the applied treatment as well, thus providing diagnostic information for therapeutics of benign and malignant tumors, encouraging a need to re-conceptualize how the efficacy of therapies is investigated by studying the viscous and elastic mechanical properties of healthy and cancerous brain tissues and designing materials that appropriately mimic these mechanical features [17,20,21]. Accordingly, the expansion of knowledge on tissue ECM properties and its impact on cellular biology is required for the development of adaptable in vitro models of hardly available tissues, such as brain tissue. In this review, we summarize the current knowledge on the mechanical properties of the brain and discuss the implications of these features in the effectiveness of drugs aimed at treating central nervous system cancers.

## 2. Composition and Biological Meaning of Brain’s Extracellular Matrix

**Figure 1:**
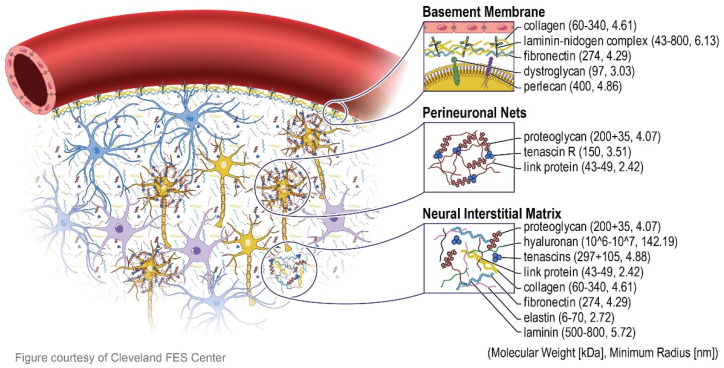
The composition of major compartments of brain ECM. The ECM in the brain is composed of three primary components, namely the basement membrane (basal lamina) that surrounds the cerebral vasculature, the perineuronal net which lies around neuronal cell bodies and dendrites, and the neural interstitial matrix that is diffusely spread between parenchymal cells. The yellow, purple, and blue cells represent neurons, microglia, and astrocytes, respectively. All of the proteins that make up the ECM have a diameter of tens to hundreds of nanometers, providing inspiration for the next generation of nano-architecture-style cerebral electrodes. Figure adapted with permission from Ref. [24]. 2018, Kim U.

In terms of biomacromolecules composition, the brain ECM is comparable to the ECMs of other tissues [22]. However, several of its elements are specifically found in the brain (Figure 1). Furthermore, each brain area has a distinct microenvironment and expresses various ECM proteins [22,23,24]. These characteristics should be considered in in vitro models mimicking brain neuronal tissue, since they appear to have unique consequences for neuronal functionality.

**Figure 2:**
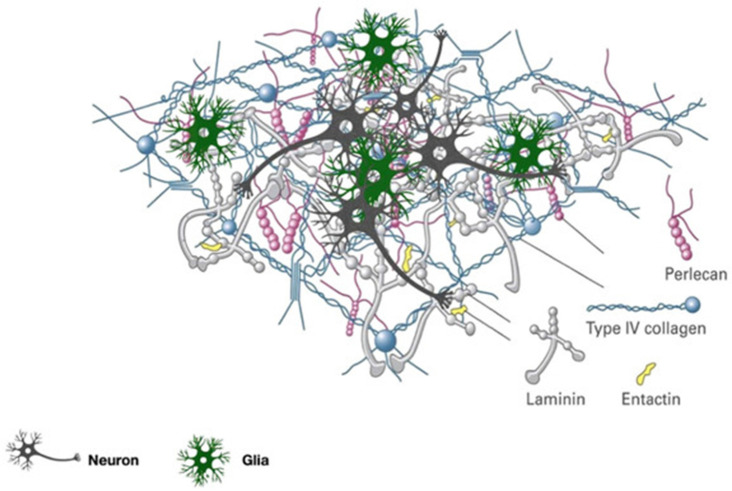
The basal lamina network is depicted schematically. Both laminin and collagen IV form a network resembling a sheet. Entactin and the perlecan complex operate as a connection between these two networks. Laminin, in combination with collagen IV, promotes cell attachment, differentiation, migration, and proliferation. Additionally, type IV collagen and laminin, in addition to fibronectin, are involved in the creation of tight junctions. Perlecan acts as a cross-linker between laminin and collagen IV, hydrating the matrix and contributing to the basal lamina’s selective filtering characteristics. In comparison to those structurally significant components, the loss of the tiny cross-linking molecule entactin appears to have a smaller effect on the structure and function of the basal lamina. Figure adapted with permission from Ref. [39]. 2020, Rauti R.

Most basically, the brain’s ECM biomacromolecule composition consists of: (i) glycosaminoglycans (GAGs)–hyaluronic acid (HA) [17,22,25]; (ii) proteoglycans (PGs)–chondroitin sulfate proteoglycans (brevican, versican, neurocan, and phosphacan) [22,26,27,28,29,30], and heparan sulfate proteoglycans (syndecan, glypican, agrin, and perlecan) [25,31,32,33] and (iii) glycoproteins—link proteins, tenascin-R, collagens, fibronectins, laminins, and nidogens [3,17,32,34,35,36,37,38] (Figure 2). The scaffolding for the ECM structure is made up of GAGs, namely HA polymers. Notably, many of the biological functions of HA in the ECM are due to protein’s high water-binding capability. These functions include supporting cell migration and preserving the ECM’s integrity by interacting with other matrix proteins [22,25]. In the central nervous system (CNS), the two primary types of PGs are chondroitin sulfate proteoglycans and heparin sulfate proteoglycans [25,31]. The most prevalent chondroitin sulfate proteoglycans are lecticans, which are involved in gliogenesis in the developing brain, tissue repair after brain injury or in brain tumors, neuronal adhesion, axonal growth, development of the nervous system, and inhibition of neurite outgrowth [25,31,32,33].The other type of PGs are involved in binding different proteins, basement membrane association, signaling, structural functions, growth factor signaling and sensitivity, binding proteins during neuronal development, and organization of the basement membrane [25,31,32,33]. Glycoproteins are engaged in interactions between HA and aggrecan, formation of lectin complexes, antiadhesive and adhesive functions dependent on cell type, expression in tumors, vascular basement membrane, cell development and differentiation, and creation of strong complexes [3,17,22,32,34,35,36,37,38].

Importantly, extracellular matrix composition and its nanomechanical features affect the mechanosensitive molecules that modulate the biological response of brain cells, thus displaying an impact on CNS tumor development. Among a spectrum of transmembrane proteins that are able to undergo structural and functional modifications in response to mechanical stimulation, integrins, G-coupled receptors (GPCRs), YAP/transcriptional co-activator with PDZ-binding motif (TAZ), cadherins, ion channels, and growth-factor receptors are the most important [40,41,42,43,44,45,46]. Particularly, integrins and integrin adhesion complexes (IACs) formed by them due to their ability to transfer force between the ECM and cytoskeleton are the leading molecules for mechanotransduction. Activation of integrins leads to cytoskeleton modifications and the activation of genes involved in cell proliferation, invasion, and survival [47]. Importantly, integrin expression is elevated in a variety of malignancies, including glioblastoma [48]. Regardless, the αvβ3 and αvβ5 integrins were the first ones discovered to be differentially expressed in gliomas vs. normal brain tissues, in the most angiogenic and invasive subtypes of CNS cancers, as mesenchymal GBM subtype, a global overexpression of integrins, was reported [48]. Additionally, studies on GBM have shown that expression of αvβ3 is associated with a worse prognosis and a shorter time to progression, as well as resistance to anticancer treatment, including temozolomide [49,50]. Importantly, in some cancers, such as breast cancer, higher levels of β1 integrin, active focal adhesion kinase (FAK), and active AKT are found along invasive fronts, and correspond with increased stiffness of tissues [51]. Similar to integrins, G-coupled receptors, including epidermal growth factor receptor (EGFR), are continuously internalized from the cell surface and display a considerable impact on CNS tumors development. Research performed by Pang et al. not only demonstrated that in glioma cancer stem cells, EGFR expression is elevated, but also prove that its activity correlates with decreased sensitivity to radio and chemotherapy. Accordingly, in some studies, EGFR inhibition was noted to reverse this tendency and improve the treatment efficiency [52]. Nevertheless, some clinical trials using EGFR inhibitors are disappointing, which might be partially determined by the neglect of nanomechanical aspects of brain tissues [53]. Particularly, it was established that mechanosensitive integrins account for partial activation of EGFR signaling pathways, leading to promotion of ERK/PI3K pathways and stimulation of cellular growth in the following steps, even in the absence of other growth stimuli [44]. Importantly, Umesh et al. revealed that a stiffness-associated increase in glioma cells’ proliferation depends on activation of EGFR; as demonstrated, enhancing the microenvironmental stiffness results in augmented EGFR/Akt expression, phosphorylation, and inhibition of EGFR-associated pathways, and weakens stiffness-mediated cellular effects. For this reason, simultaneous administration of mechanotransduction-affecting agents and EGFR inhibitors is recognized as a potential approach to enhance the activity of both therapeutics [43].

Apart from integrins, which are the most essential cell adhesion molecules (CAMs) when it comes to mediating cell–matrix adhesion, cadherins also contribute to tumor infiltration and spread. Cadherins assemble into subtype-specific, adherent intercellular junctions, primarily through homophilic interactions. Since various cadherins are involved in cell sorting, it is believed that cells expressing a certain cadherin subtype preferentially attach to cells expressing the same cadherin subtype [54]. Nonetheless, the expression of cadherins in cells may also be heterogeneous, with cells expressing numerous cadherin subtypes, resulting in cadherin-mediated heterotypic adhesion [55,56]. Cadherins are abundantly expressed in the nervous system, where they facilitate cell–cell connections within neural networks and dynamically contribute to the growth and function of neurons [57]. In malignant gliomas, cadherin levels fluctuate, as evidenced by multiple investigations, and reports in this respect are contradictory [58,59,60,61]. Some findings demonstrate an inverse relationship between N-cadherin expression and glioma invasion [62], while others demonstrate no link [61], or indicate a positive correlation with the grade of the gliomas [59,61].

Another mechanosensitive protein involved in controlling the biological processes in the brain—YAP—binds to transcription factors in the nucleus, governing the invasiveness and chemoresistance of cancer cells [63]. In one study, expression of YAP1/TAZ and their target gene, BIRC5, was positively correlated with the prognosis of glioma patients, with subsequent downregulation of large tumor suppressors 1/2 (LATS1/2)-associated pathways [64]. This observation was confirmed by Guichet et al. [65]. In one of the studies, the authors analyzed the expression of YAP1 in 117 clinical samples of glioma, as indicated by the 2016 WHO classification, and demonstrated that YAP1 is strongly correlated with glioma molecular subtypes and patient prognosis. Moreover, this finding was verified using a separate TCGA database cohort; as reported, YAP1 should be recognized as a biomarker in predicting patient survival, particularly in low-grade gliomas [65]. In another study, using both a cell culture model and a xenograft mouse model of GB, it was evidenced that differential YAP expression in glioma cells promotes tumorigenesis and results in the clonal dominance of YAP-expressing cells, showing that competitive interactions between diverse tumor cells promote carcinogenesis in CNS [66]. Importantly, the nucleus/cytoplasm distribution of YAP is responsive to mechanical stimuli experienced by cells, including substrate stiffness, cytoskeleton tension, nuclear deformation, and extracellular mechanical tension/compression [67]; however, the molecular mechanisms by which YAP reacts to mechanical stimuli are currently under investigation.

Finally, mechanosensitive ion channels, including PIEZO channels, should be taken into consideration when analyzing the microenvironment of CNS in the aspect of brain tumors development. Induction of physical force via membrane tension allows Na^+^, K^+^, and Ca^2+^ to permeate via PIEZO channels [68]. One study by Chen et al. reported that the mechanosensitive ion channel PIEZO1 is overexpressed in aggressive gliomas and regulates tumor growth [69]. Furthermore, it localizes at focal adhesion and interacts with integrin–focal adhesion kinase signaling, which regulates glioma malignancy and stiffness [69]. Further research by Zhou et al. into PIEZO1 demonstrated those channels as a potential prognostic marker in glioma subjects. Accordingly, PIEZO1 expression was correlated with glioma malignancy, and it was evidenced that cancer progression occurs due to involvement of PIEZO1 in multiple signaling pathways affecting cell proliferation and microenvironment [70]. Moreover, it is also widely recognized that other ions channels (e.g., K^+^, KCa3.1, KCa1.1) are involved in glioma malignancy, since the management of ion flow and the accompanying modulation of water flux are crucial for migration and invasion. Moreover, signaling pathways influenced by ion channel activity play important roles in cell survival and proliferation [71].

Apart from above-mentioned mechanosensitive molecules, metalloproteinases (MMPs) are one of the prevailing enzymes in the extracellular environment structure, and their expression and activity were reported to be variable in CNS tumors. The most crucial function of MMPs is the degradation of most extracellular matrix proteins, allowing the angiogenesis to occur. Exuberant and atypical vasculature is one of the distinguishing histological features of GB. Matrix metalloproteinase 9 (MMP-9) studied by Xue et al. showed upregulation in glioma tissues, and its expression was directly correlated with WHO tumor grading. Furthermore, it was also noted that overexpression of MMP9 expedited tumor growth and generated a considerable increase in clonogenic capacity [72]. Zhou et al. also correlated MMP-9 and MMP-2 with tumor grading of primary and recurrent gliomas [73]. Moreover, increases in expressions of MMP-9 and MMP-2 were indicative of a poor prognosis in glioma. Another group evidenced MMP-2 correlation with glioma and found that expression of MMP-2 was significantly correlated with tumor diameter and grade [74]. Furthermore, Kasten et al. tested MMP-14 as a biomarker for imaging glioma with successful effect in mice models using positron emission tomography imaging [75]. Moreover, MMP-2 and MMP-9 have a synergistic impact on the breakdown of endothelial basement membrane in gliomas and facilitate the release of ECM-bound VEGF [76]. Those reports strongly confirm the role of MMPs in shaping the pro-tumorigenic microenvironment. Notably, expression of MMPs is also regulated by mechanical stimuli, as evidenced by reports using various cancerous and non-malignant cell culture models. For instance, using vascular smooth muscle cells, Seo et al. demonstrated that platelet-derived growth receptor- β (PDGFR-β), as a cell surface mechanoreceptor, transmits mechanical signals to intracellular sensors to produce MMP-2 via regulation of Akt activity, indicating a crucial role in vascular remodeling induced by mechanical stress and linked to arterial hypertension [77]. On the other hand, by employing fibronectin-coated polyacrylamide gels to modify substrate stiffness without modifying ligand density (as model of liver fibrosis), it was demonstrated that fibrotic rigidities downregulate MMP-9 expression and secretion and upregulate TIMP-1 secretion [78]. Importantly, such mechanical-affected variations in MMPs expression affect not only the angiogenesis processes, but also cause further alterations in the subsequent steps of the elasticity parameters of tissue, and vice versa. For instance, at the locations of matrix deterioration produced by MMP, the matrix becomes more rigid, which results in stiffness gradients and localized stiffening of ECM, both of which are needed for the creation of new blood vessels [79]. Nevertheless, when the extracellular matrix of tumor tissue stiffens, VE-cadherin cell–cell junctions are destroyed, which compromises vessel integrity and leads to leakage [80]. Due to the vasculature’s permeability and malformation, as well as the pressure applied by the tumor’s solid stress, the interstitial pressure and blood flow rise, generating fluid stress in the tumor tissue [81]. In addition, VEGF expression directly induces the deposition of laminin matrix, resulting in a local stiffening of the extracellular matrix and the formation of a stiffness gradient [82,83]. Based on these reports, attempts to develop potent antiangiogenic therapeutic for glioma patients are carried out. Among approaches tested, the antiangiogenic therapies using anti-VEGF agents, including bevacizumab and cediranib, are the most accepted and suggested to improve the survival of cancer patients [84,85,86,87,88,89]. Interestingly, reversal of abnormal mechanics of brain tissues was presented as a marker of treatment efficiency, according to one of the recent studies [90]. When analyzing the effects of anti-VEGF antibody effectiveness against glioblastoma, Schredel et al. used MRE to demonstrate that the reduction in the viscoelasticity and phase angle in GBM was mitigated in treated animals, which might be attributed to normalized vasculature and improved myelin preservation within treated tumors [90]. Overall, the sum of these reports strongly encourages the recognition of nanomechanical aspects of tumor tissues as crucial not only for exploring the biological behaviors of cells, *per se*, but also for a deeper understanding of cascades of processes leading to development of malignancies.

## 3. Nanomechanical Properties of Brain Tissue and Their Significance in Health and Disease

Previous studies performed using mostly liver and renal tissues described that variations in tissue mechanics are frequently associated with pathological abnormalities observed in tissue histology, such as vascularization of tissue ECM and collagen accumulation [91,92,93]. Importantly, it has been reported that early stages of disorder development correlate with alterations in cell/tissue stiffness, and these often occur before any changes are detected via histopathological analysis [94,95]. The majority of research aimed at understanding mechanical characteristics of tissues that formed human CNS was carried out using brain tissues with the goal of determining its stiffness, viscosity, and intracranial pressure, and in part, they relied on non-invasive magnetic resonance elastography (MRE) and tissue rheology [18,96,97].

**Figure 3:**
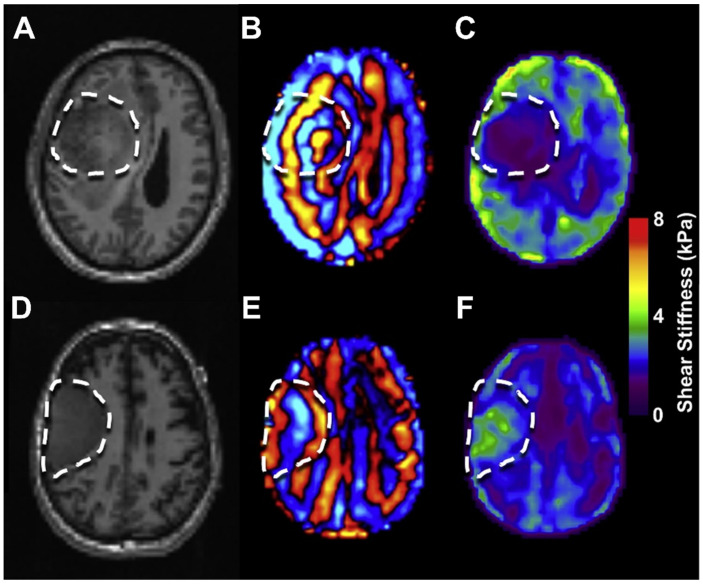
Tumor stiffness differences for two patients with meningiomas. T2-weighted anatomical images (**A**,**D**), curl wave images (**B**,**E**) and elastograms (**C**,**F**). The first patient (**A**–**C**) had a substantially softer meningioma than the second one (**D**–**F**). In the first example, the shear wavelength is much shorter than in the second one. Figure adapted with permission from Ref. [108]. 2015, Pepin K.

MRE is a magnetic resonance imaging (MRI)-based technique used for quantitatively imaging the mechanical properties of tissues in real time [98]. Its clinical value relies on its non-invasiveness while gathering data, since it does not require a biopsy or surgery. The quantitative metrics that may be recorded are: loss modulus, storage modulus, damping, viscosity, dispersion, anisotropy, non-linearity, and the functional mechanics of the brain [99,100] (Figure 3). However, technical challenges arise as a result of frequency dependency, which generates varied mechanical responses in the tissue and makes data comparison between laboratories difficult [101]. Over a decade ago, the first brain MRE investigations were published, with the goal of establishing reference values for the global stiffness of a healthy adult human brain and preliminary tumor results [102,103,104,105]. Recently, MRE began to receive attention due to increases in resolution required to adequately investigate brain structures [106]. Despite early studies demonstrating a lack of correlation between published properties, researchers have recently discovered highly reliable property metrics and similarities between a typical set of properties in the healthy brain, even when using different experimental methodologies and reported viscoelastic properties [99,107].

Most basically, glioblastomas were found to be softer than healthy brain parenchyma, as reported by Streitberger et al. in 2014 [109]. Simon et al. confirmed this discovery through comparison of three GBs with contralateral parenchymal stiffness and demonstration of a wide variation of mechanical properties amongst different cancer types [110]. In another research study, glioblastomas were softer than brain parenchyma in 18 patients, but grade IV gliomas were also much softer than grade II gliomas, according to Pepin et al. [111]. Despite gliomas being uniformly softer than brain parenchyma, other tumor forms were more diverse, including soft and hard areas, as demonstrated by Reiss-Zimmermann et al.; however, they stated that meningiomas could be clearly distinguished from glioblastoma, anaplastic astrocytoma, intracerebral abscesses, and cerebral metastasis [112]. Sakai et al. studied 34 patients with different histological diagnoses of brain tumors and reported that meningiomas were harder than pituitary adenomas, which was also correlated with intraoperative consistency grading. Firm tumors had higher maximal shear stiffness, but not higher mean shear stiffness than non-firm tumors [113]. Murphy et al. reported correlation between mean shear stiffness of meningiomas with surrounding brain to surgeons’ qualitative evaluation of tumor stiffness in 12 cases [114]. Notably, Hughes et al. confirmed these results of mean shear stiffness with surgeons’ impression and durometer measurements [115]. Specificity, sensitivity, and predictive values of mean shear stiffness for meningioma heterogeneity, homogeneity, and hardness were high. Hughes et al. also studied pituitary adenomas [116]. Studies showed that values of soft tumors were lower in comparison to intermediate tumors, which were evaluated intraoperatively. In 2018, Weickenmeier et al. studied the differences between human and pig brains, and concluded that human brains are softer and less viscous than deceased pig brains [117]. Leading up to clinical application, it will be necessary to understand the processes behind these mechanical changes, as well as to conduct large-scale clinical research investigations and methodology standardization [118].

**Figure 4:**
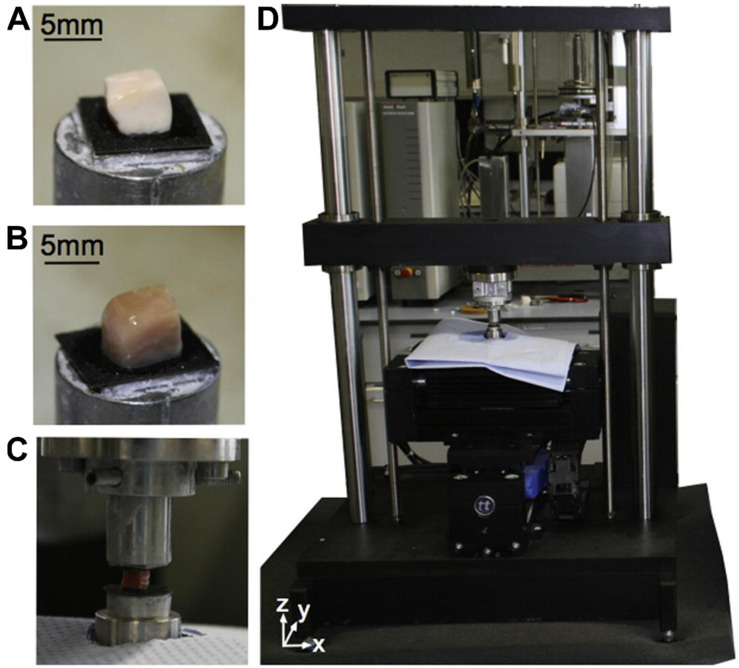
Rheological measurement setup. (**A**) White matter sample; (**B**) gray matter sample; (**C**) sample glued to upper and lower plates; (**D**) hydrated sample mounted into device. Figure adapted with permission from Ref. [120]. 2017, Budday S.

The other approach to characterizing nanomechanical parameters of brain tissues is to explore the rheological features (Figure 4). It is well established that the rheological behavior of the human brain is important to brain function and failure [119]. However, the heterogeneous microstructure of brain tissue, in which cell composition and morphology change from one region to the next one, makes characterization challenging [120]. Moreover, while early research focused on large samples of various tissues [121,122,123], more recent studies have attempted to quantify regionally changing tissue stiffness, with mixed outcomes: some researchers found cortical gray matter to be stiffer than white matter, while others published the opposite [124,125,126,127,128,129].

Overall, there is still much to learn about the mechanical behavior of brain tissue as a whole, and only a limited number of people have studied human brain tissue due to the fact that it is not easily accessible [101,120,121,122,124,127,129,130,131,132,133,134,135,136]. In early research on rheological properties of the brain tissue, Shuck et al. showed the differences between gray and white matter [121]. However, the differences were considered small enough that the average values for white and gray matter were used in the reports and model fitting. In another study, Galford et al. tested human and monkey brains and observed that humans have softer and less viscous brains, but it is worth mentioning that the rhesus monkey was measured within 1 h after sacrifice and human brains were tested 6–12 h after death [132]. Thus, time after death might alter the data recorded for the human brain. On the other hand, Donnelly et al. reported that they did not observe significant dependence based on sample location; however, they discovered significant variations in samples between different subjects [122]. Prange et al. correlated these results with their own research and with the use of fresh human tissue, which is considerably less stiff than the previously researched autopsy data; thus, it is far more comparable to fresh porcine data than to human autopsy data [130]. Franceschini et al. came to the conclusion that brain tissue mechanical modeling should involve a porosity model, fluid-saturated, non-linear solid with extremely low volumetric compressibility [133]. Importantly, most of the research has been carried out on adult brains. To address this issue, Chatelin et al. tested the rheological properties from 2-month-old to 55-year-old subjects [131]. Between the ages of 5 and 22 months, a considerable rise in both loss and storage moduli were seen and confirmed statistically, which reflects the change in water content of the tissues. Furthermore, adult brain appears to be 3–4 times stiffer than that of young children. Likewise, it was discovered that the brainstem is roughly 2–3 times stiffer than both white and gray matter from the thalamus and corona radiate. Chatelin also suggested that further research should concentrate on a larger sample size and on 1-year-old children for implementation of realistic mechanical properties into finite element models [131]. Jin et al. performed compression, tension, and shear tests on a total of 240 brain tissue samples and concluded that white matter was stiffer than gray matter in both shear and compression, but also recorded regional difference in compression and tension [129]. Moreover, directional dependence was observed for white matter under shear loading. Non-linear and viscoelastic response was in agreement with previous research [129,133] and confirmed by Budday et al. [127]. Mechanical properties were found to be region dependent, with overall brain tissue also being time dependent. Budday also showed improvements in finite viscoelastic Ogden-type models for human brain tissue which were closer to experimental data [120,124]. Forte et al., on the other hand, reported that sample size differences are negligible in terms of different testing conditions [134]. However, temperature and humidity play a major role in rheological analysis due to the duration of the test. In that case, white matter can suddenly start to stiffen, and further increases in temperature enhance the drying process, leading to a reported storage modulus up to almost 22 times higher, if humidity cannot be kept at high levels. A comparison of healthy brain and glioma tissue was performed by Tabet et al. [136]. The storage modulus of healthy tissue at 1 rad s^−1^ was 189 Pa, and tumor tissue at the same frequency reached 536 Pa. These differences were used to create dual network bioelectronics hydrogels that can be tuned and used in future neural stem cell models. Alternatively, patient-derived xenograft models with readily adjustable mechanical properties were used as drug-delivery reservoirs for improved drug bioavailability for glioblastoma tumors, which was reported by Parkins et al., and they showed a potential to improve the survival of glioblastoma patients [135]. Due to limited availability and due to the significant degree of resemblance between animal and human brain tissue in terms of anatomical traits and mechanical characteristics, animal brain tissue is often employed as a substitution for human brain tissue [123,137].

**Figure 5:**
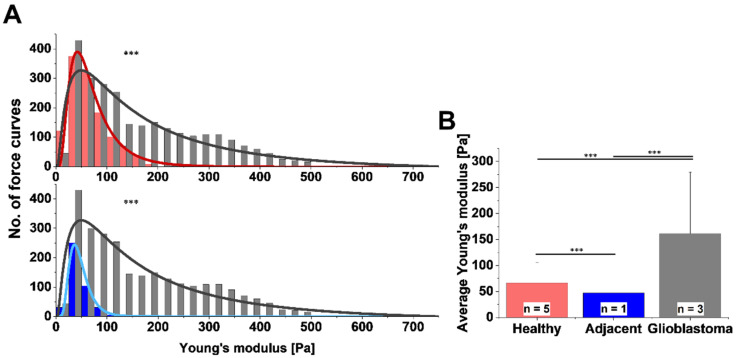
Variation in nanomechanical properties among different brain tissues. AFM-measured (**A**) distribution and (**B**) mean values of Young’s modulus for healthy brain tissue (red), tissue adjacent to GB (blue), and GB tissue (gray). Statistical differences were measured using Kolmogorov–Smirnov test and unpaired Student’s *t*-test. *** *p* ≤ 0.001. Figure adapted with permission from Ref. [16]. 2020, Cieśluk M.

If nano-mechanical-scale resolution is needed, atomic force microscopy (AFM) can be used [138]. AFM is suited to detecting stiffness fluctuations within tissue samples, and its nanoscale resolution allows examination of cellular and extracellular elements, as well as comparison with tissue histology [16,139]. Depending on the experimental setting, nanomechanical properties can be assessed at different indentations with various force velocity, different cantilever stiffness, tip geometry, and working modes. Still, it is mostly the elastic behavior of the sample that can be characterized based on these measurements. Our data acquired using AFM to assess tissues stiffness clearly present the differences between the stiffness of grade IV glial tumors in comparison with healthy tissue (obtained during surgery from the healthy tissues sample extracted to gain access to remove the tumor), while adjacent samples had similar stiffness to the healthy tissues (Figure 5) [16]. Moreover, the stiffness distributions of malignant tissues are highly heterogeneous, indicating the inhomogeneity of the examined cancer samples [16].

Other researchers reported an increase in surface roughness in stroke-affected brain tissue, which was created by the increased tension in the brain tissue [140]. On the other hand, Bouchonville et al. observed that the pituitary gland had intermediate stiffness (geometric mean of 9.5 kPa) in comparison to other organs [141]. Furthermore, pituitary gland tissue was highly inhomogeneous at cell scale and recorded local changes in stiffness of the tissue indicate kPa/µm gradients of rigidity at micron scales. Minelli et al. were the first to register nanomechanical characterization of human brain abscess tissue with AFM [142]. Their group measured a three-layered structure with layer 4 (inflammatory border) being softest at around 10 kPa and layer 3 (collagenous capsule) being stiffest at 1.04 MPa, which is consistent with its function. The average stiffness of the three-layered structure is greater than that of the surrounding peri-abscess tissues, which exhibit a mean E of 1.4 kPa, consistent with the abscess’ mechanical compression of the surrounding tissues. Moreover, Minelli stated that these layers present a typical viscoelastic behavior, which is controlled by viscous and dissipative forces. Ciasca et al. discovered a significant difference in the biomechanical response of glioblastoma tissues compared to normal peritumoral areas (E of 1 kPa for normal peritumoral white matter, 0.3 kPa for necrosis in glioblastoma, and 10 kPa for non-necrotic tissues) [143]. Furthermore, Ciasca stated that these results may have actual use in clinical practice, and can open up new opportunities for optimization of the tumor resection area. AFM has been effectively employed in other research on brain tumor tissue to better comprehend the mechanopathology of this lethal illness, according to reports [144,145,146,147]. Nonetheless, fixed or frozen human brain samples have mostly been employed to date. AFM may be used to detect stiffness changes in tissue samples, and its nanoscale resolution enables evaluation of cellular and extracellular tissue components that may be compared to fluorescence imaging, histopathological staining, or Raman spectroscopy analysis [143,144,145,146,147,148]. Due to previously mentioned difficulties regarding access to fresh samples of human brain tissue, particularly for healthy, untransformed tissue, the majority of research is conducted on animals or human brain tumors, with a normal rat or mouse brain serving as a control [125,149,150,151].

Moreover, AFM’s ability to image delicate materials in air and liquid without fixation or inflicting significant damage makes it a potent instrument for analyzing biological samples, including exosomes. Exosomes contribute to the growth and spread of cancer by transporting bioactive chemicals between cancer cells and other cells in the microenvironment [152]. Malignant gliomas emit a disproportionately large amount of exosomes, which may facilitate their invasion, metastasis, and proliferation [153]. Interestingly, glioma-derived exosomes may diffuse into body fluids, including blood [154] and cerebrospinal fluid (CSF) [155], by traversing the blood–CSF barrier (BCSFB) and the blood–brain barrier (BBB), and for this reason, glioma-derived exosomes have been suggested as useful biomarkers to follow the course of glioma [156]. A nanoscale investigation of exosomes performed by Thakur et al. demonstrated that hypoxic GM-derived exosomes have distinctive physicochemical features that facilitate their absorption by endothelial cells, such as increased roughness, decreased elasticity, and higher adhesion force [157], revealing that these markers might be used to detect the hypoxic and malignant cells. A nanoscale analysis also was used to demonstrate that glioblastoma-derived exosomes (and potentially also exosomes from other malignancies) are characterized by the presence of surface nanofilaments, allowing them to be uptaken significantly more efficiently when compared to normal cells [158].

## 4. The Influence of Brain Tissues’ Nanomechanical Properties on Drug Effectiveness

Since it is well recognized that tissue stiffness is known as a critical determinant of cellular function and behavior, there is a need to develop in vitro models that precisely capture physiologically relevant mechanical conditions, including stiffness and viscosity of brain tissues. Moreover, the understanding of the role of mechanics in cell function and response to drug treatment is crucial for new mechanistic-based approaches to guide the development of more effective cancer treatments. Notably, one of the most crucial issues when novel therapeutics are tested is the interference between results obtained in vitro and using in vivo models. Despite remarkable anticancer effects in preclinical experiments, most compounds fail during clinical trials, since they are tested using preclinical systems that do not account for the mechanical properties of the extracellular environment. At the same time, new therapeutic options are required due to the high mortality rate caused by neoplastic diseases of the nervous system, particularly gliomas, on which the majority of research is focused.

**Figure 6:**
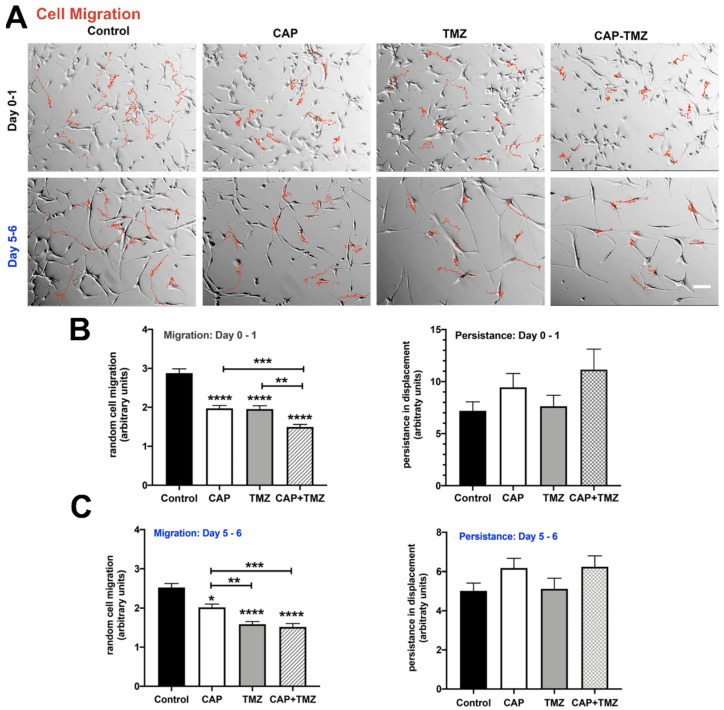
The impact of cold atmospheric plasma (CAP) on cell migration. (**A**) Control (untreated), CAP-only (180 s), TMZ-only (50 μM), and combined CAP–TMZ treated cells. Red indicates the trajectories of 10 representative cells evaluated during a 16 h period on Day 0–1 and Day 5–6. Images were captured every 10 min until a total of 100 images were acquired. The CAP-TMZ condition results in shorter cell migration paths than the control variables (**B**,**C**). The graphs illustrate the velocity and displacement of 60 cells per variable. Persistence was calculated as the ratio of net displacements to total displacements. Unless otherwise specified, error bars represent the standard error of the mean, and an asterisk denotes statistical significance relative to untreated control. Scale bar: 14 μm. Statistical significance was determined as: * *p* < 0.05; ** *p* ≤ 0.01; *** *p* ≤ 0.001, **** *p* ≤ 0.0001. Figure adapted with permission from Ref. [204]. 2020, Gjika E.

More accurately, gliomas account for approximately 80% of all tumors arising in the central nervous system, with incidence of about 7 per 100,000 persons worldwide, among which glioblastoma (WHO Grade IV) is recognized as the most common and deadly [159]. Moreover, GBs pose a challenge in neuro-oncology due to their infiltrative nature, resistance to apoptosis, tendency for reappearance, and resistance to standard therapies [160]. Regardless of the improvement in diagnostics, surgery, and additional chemotherapy, the prognosis remains unfavorable, with a median survival of 7–18 months [161,162,163]. Currently, the standard treatment for glioblastomas is surgery [164]. To date, a significant number of chemotherapeutic agents have been suggested as possible treatment of glioblastomas, including alkylating agents (carmustine, procarbazine), inhibitors of topoisomerase I and II, taxanes, and anti-VEGF inhibitors; nevertheless, their impact on survival was limited and unclear [165,166,167,168,169]. Some hopes are associated with employment of temozolomide (TMZ), a lipophilic, orally obtainable monofunctional DNA alkylating agent of the imidazotetrazine class, as an antineoplasm agent in glioblastoma treatment [170]. TMZ is unstable at a pH above 7 [171,172]. Fortunately, brain tumors have more alkaline pH compared to healthy tissue, which helps with TMZ activation within tumor tissue, increasing its effectiveness in brain tumor therapy [173,174]. Beneficial results and improved progression-free survival in cases of recurrent glioblastoma were presented recently for bevacizumab (Avastin), a humanized monoclonal antibody that binds to vascular endothelial growth factor (VEGF) ligand, inhibiting further activation of its receptors [175]. It is noteworthy that the combination treatment of bevacizumab with radiation and TMZ for therapy of newly diagnosed glioblastoma shows some promising results, but is also associated with higher rates of toxicity [87,176,177]. Lomustine is also used in recurrent glioblastoma and is currently being tested for primary glioblastoma, along with TMZ, with promising results for improved overall survival [178,179,180]. There is also some hope for inhibitors of EGFR, which is a transmembrane glycoprotein and is a member of the ErbB family of tyrosine kinase receptors [181]. Yang et al. provided insight into the recently FDA-approved antidiabetic medication, which may have anticancer properties and synergistic effects with TMZ treatment, and that could enhance chemotherapy efficacy in glioblastoma [182]. Synergistic effect was also showed in other research papers [183,184,185]. Integrins infrequently mutate in cancers [48]. They may not function as oncogenes, but may interact with them to promote carcinogenesis, either by expression levels and/or location in tumor cells relative to normal cells, through post-translational changes, or through integrin recycling [186,187]. Indeed, changes in the integrin pattern are often related to tumor growth in a variety of malignancies [49,186]. Integrins provide intriguing targets for cancer therapy due to their role in tumor cell activities, their interaction with multiple pathways, and their membrane location. Furthermore, Christmann et al. reported that silencing integrin αVβ3 sensitizes malignant glioma cells to TMZ [188]. Zhang et al. also showed that integrin is a promising diagnostic target that leads to in vivo detection with high target-to-background ratios and low background fluorescence [189]. Shaim et al. suggested the αv integrin/TGF-β axis as a potentially useful therapeutic target against glioblastoma stem cells due to improvements in the function of natural killer cells [190,191]. Apart from integrins, the MAPK/ERK pathway has been implicated in numerous malignancies, most notably melanoma, as a frequently dysregulated system [192]. Coordinated targeting of this pathway may have a synergistic impact on tumor growth control [193]. Clinical studies using a variety of MEK inhibitors, including trametnib, cobimetinib, and CI 1040 (PD184352), have shown that some melanomas, particularly those with BRAF mutations, reduce size. Additionally, the MEK inhibitor PD0325901 has been shown to be beneficial in melanoma cell line regardless of BRAF status [86,194,195,196,197]. PD0325901 has been shown to be effective in vitro and in vivo when regulating tumor development in animal models of glioblastoma, while some research indicated some difficulties with restricted access across the blood–brain barrier [198,199]. Inhibition of transport proteins, which are present at the blood–brain barrier, may be a valid target for improved brain penetration without dosage alteration [200]. In another study, cold atmospheric plasma (CAP) has repeatedly shown anticancer efficacy when used alone, and is now being considered in conjunction with temozolomide [201,202,203,204]. The CAP approach has shown specificity for a variety of cancer forms, including glioblastoma [205,206,207]. However, the precise therapy criteria for glioblastoma have not been determined [208] (Figure 6).

**Figure 7:**
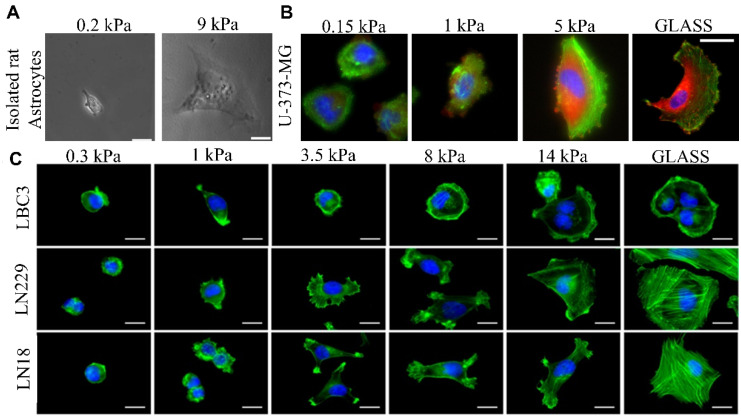
Gel stiffness influence on cells spread area. Phase images of isolated astrocytes on soft and stiff polyacrylamide gels (**A**). Human glioblastoma cells U-373-MG on hyaluronic acid hydrogels of various stiffness (**B**). Isolated human glioblastoma cells LN18, LN229 and LBC3 on various polyacrylamide gels (**C**). Scale bars: A, B—50 µm; C—15 µm. Figure adapted with permission from Refs. A—[219]. 2006, Georges P.C.; B—[220]. 2011, Ananthanarayanan B.; C—[17]. 2017, Pogoda K.

Although a spectrum of novel antiglioma therapeutics is presented, only a limited number of them are tested using experimental settings with variable nanomechanical parameters. Importantly, the research which was performed to date demonstrated that the mechanical properties of ECM and cell–cell contact influence the cell response, including remodeling of their cytoskeleton structure causing changes to the spread area or the strength of cell adhesion to the substrate [209]. Many different cell types modify their morphology when seeded on substrates with various stiffness [210,211,212]. It is known that cells grown on stiff substrates form actin stress fibers, increase content of cytoskeletal proteins and integrins, and enhance signaling pathways corresponding to retractility, thus exhibiting a more widely spread phenotype than when grown on soft substrates (Figure 7) [212,213,214,215,216,217,218].

**Figure 8:**
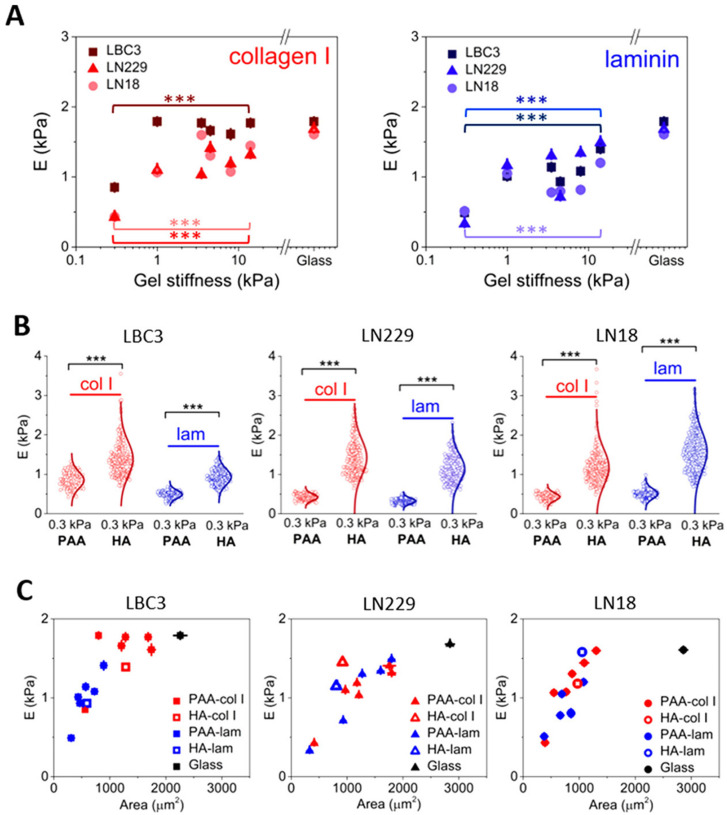
Regulation of glioblastoma cell rigidity by substrate composition and stiffness. (**A**) Cellular stiffness as a function of substrate stiffness for collagen I or laminin-coated PAA hydrogels. (**B**) Variations in the cell stiffness when PAA is replaced by HA. (**C**) The stiffness of GB cells as a function of their spreading area. The unpaired Student’s *t*-test was used to confirm significant differences in cell stiffness between in panels A (between 0.3 and 14 kPa PAA gels) and B (between 0.3 kPa PAA and 0.3 kPa HA); denotation: ***, *p* < 0.00). Figure adapted with permission from Ref. [17]. 2017, Pogoda K.

Interestingly, according to the studies carried out so far, it appears that not all cell types show the substrate stiffness effect. However, the response from mechanosensitive cells is alike, and the cell types adhere and spread more to stiffer substrates. Focal adhesion complexes and associated proteins are involved in the formation of traction forces and cytoskeletal structure necessary for adherence to substrates on soft materials [210,211,212,213]. Nevertheless, it has been documented that not only do the differences in substrate stiffness influence the spread area of cells [18], but also the change in adhesive ligands from collagen I to fibronectin or laminin can influence the spread, or even the cell stiffness itself [17,18,221]. Thus, a new area of research should focus on the influence of substrate stiffness and adhesive ligands on the cell’s response to anticancer drugs [222,223] (Figure 8).

Additionally, an increasing number of publications show that cell cycle progression or proliferation capabilities might be influenced by substrate mechanical qualities. The first was reported in smooth muscle cells, fibroblasts, mammary epithelial cells, and other non-neuroglial cells, demonstrating that pathological ECM remodeling and stiffening of the microenvironment promotes cell cycle progression via integrin-dependent signaling to FAK, cyclin D1, and Rac [224]. Other publications have also shown the dependency of proliferation on substrate modifications [225,226,227]. In another study, Nicolas-Boluda et al. showed in one of the more recent publications that direct suppression of variables regulating EMC stiffness and organization may also result in enhanced treatment response. In this regard, it has been observed that inhibiting lysyl oxidase (LOS), a protein that stabilizes ECM collagen fibers, increased intratumoral T cell infiltration and migration and, in subsequent steps, also enhanced responsiveness to anti-PD-1 therapy, especially in early tumor stages [228,229]. Treatment success may also be influenced by changes in the collagen content inside tumor tissue, which has a direct effect on intracellular stiffness and motor activity [230]. As a result, changes in collagen concentration may have an influence on the efficiency of paclitaxel, ROCK inhibitors, and MMP inhibitors [230]. Thus, distinguishing between different cell responses to drugs in different environments may be critical for developing novel treatments that target particular intracellular behaviors linked with disease progression.

## 5. Summary

Despite many contradictory reports, it has been established that brain tissue stiffness parameters significantly change in the process of pathogenesis of nervous system diseases, particularly in the process of oncogenesis, which has not only diagnostic significance, but also affects the severity of the disease and, potentially, the response of cells to the applied treatment. Thus, it is necessary to recognize how nanomechanical properties determine the cellular response in order to develop better treatment for brain tumors.

## Data Availability

Not applicable.
